# Supplementary material for: DB-EAC and LSTR: DBnet based seal text detection and Lightweight Seal Text Recognition
Source: PLoS One. 2024 May 16;19(5):e0301862. doi: 10.1371/journal.pone.0301862 (PMC11098430; doi:10.1371/journal.pone.0301862)
Supplement: S1 Appendix — (DOCX) [file pone.0301862.s001.docx]

**S1 Appendix. Dataset sources.**

The dataset in this paper consists of two parts, which are categorized into Chinese electronic and real scene stamps：

The electronic seal is mainly produced by script files, drawing tools, and researchers can access these data from Table 1, or click on the links to download them directly <https://bj.bcebos.com/ai-studio-online/8f10fa9f21d3437cb1365cbaaaa500f0b162eae1555b4783b76b3cbfc505eabe?authorization=bce-auth-v1%2F5cfe9a5e1454405eb2a975c43eace6ec%2F2022-09-04T15%3A27%3A50Z%2F-1%2F%2F8751a2d8f115a391dae4c9e1ca4691ca5b32c8f441a67de89721631dd11c6b78&responseContentDisposition=attachment%3B%20filename%3Ddataset.zip>.The above datasets are open source, free to download,operated and managed by Baidu Paddle Platform.

**Table 1.URLs for the electronic seal**

| **Seal type** | **URL** |
| --- | --- |
| contract | <https://aistudio.baidu.com/datasetdetail/177475> |
| corporations | <https://aistudio.baidu.com/datasetdetail/154271> |

The Real Scenario Stamp was provided by the Nanning Municipal Audit Bureau of the Guangxi Zhuang Autonomous Region, and we believe that the Real Scenario dataset should be restricted from being made available for legal and ethical reasons for the following reasons：

Under article 280 of the Criminal Law, anyone who forges, falsifies, trades in or steals, snatches or destroys official documents, papers or seals of State organs shall be sentenced to fixed-term imprisonment of not more than three years, detention, control or deprivation of political rights, and shall also be punished by a fine; if the circumstances are serious, he shall be sentenced to fixed-term imprisonment of not less than three and not more than 10 years, and shall also be punished by a fine.

According to article 52 of the Law of the People's Republic of China on Punishments for Public Security Administration, anyone who forges, alters or trades in the official documents, papers, certificates or seals of State organs, people's organizations, enterprises, institutions or other organizations shall be sentenced to detention of not less than 10 days and not more than 15 days, and may be sentenced to a fine of not more than 1,000 yuan.

The seal is often an important identifier of the rights of the department concerned. At present, there are unscrupulous elements who take advantage of the rights of government administrative departments to seize large profits by forging seals. We believe that publicizing the real seal dataset of all government departments may be used by criminals to cause the abuse of governmental rights, generating fake seals that are more difficult to identify and causing trouble to government and enterprise offices. In fact,recognizing the authenticity of seals is also our next research direction.

Finally, in order to promote the research in the field of seal detection and recognition, related researchers can contact the corresponding author(bhhuang-66@gxu.edu.cn) or the person in charge of the intelligent office of Nanning Municipal Audit Bureau of the Guangxi Zhuang Autonomous Region (nnpzsczj@163.com),access to the full range of real data sets by providing data that verifies identity, organization, and purpose.

**Table 2. URLs for the real seal.**

| **Administrative level** | **URL** |
| --- | --- |
| district level | <http://www.gxzf.gov.cn/zcwjk/policyList/> |
| municipal level | <https://sj.nanning.gov.cn/xxgk/zcfg/> |
| other | <https://www.audit.gov.cn/gdnps/html/index.jsp> |
