# Supplementary material for: DB-EAC and LSTR: DBnet based seal text detection and Lightweight Seal Text Recognition
Source: PLoS One. 2024 May 16;19(5):e0301862. doi: 10.1371/journal.pone.0301862 (PMC11098430; doi:10.1371/journal.pone.0301862)
Supplement: Attachment — Submitted filename: Response to Reviewers.docx [file pone.0301862.s002.docx]

**Note: For the convenience of the reviewers, we have used red, green, and blue fonts for 1, 2, and 3 reviewers in “Response to Reviewer” and “Revised Manuscript with Track Changes”, respectively.**

**To reviewer #1**

This article discusses the importance of artificial intelligence in the field of seals, aiming to solve the problem of seal information localization and recognition. Therefore, an efficient DBNet-based model is proposed in this article. The paper has a certain degree of innovation, but there are also serious problems that need to be solved. Recommendations for improving the manuscript:

1. Figure 2 is incompletely plotted, for example, activation functions, normalization operations, and so on. Please plot Figure 2 carefully and add relevant descriptions.

Response：

We redrew Figure 2 and added the relevant descriptions in the second paragraph of section 2.2：“the original BottleNeck module in the ResNet network uses 1*1 convolution to adjust the number of channels and accelerate convergence via batch norm to improve generalization. Then ReLU solves gradient vanishing and promotes feature information transfer. However, BottleNeck uses 1*1 size convolution kernel and step size s is 2 for downsampling”

2. Please pay attention to the latex writing specifications for equation 10, such as "radical sign" and "superscript and subscript". Because the symbol "^" is often represented as an XOR operation in computer languages such as Java, C++, and Python.

Response:

We strongly agree with and adopt your suggestion.Here is the new formula 10 in section 2.3.

$$Attention\left( Q,K,V \right)=Softmax\left( \frac{QK^{\text{T}}}{\sqrt{d_{k}}} \right)V$$

3. In Section 3.2, the article mentions biformer, an attention mechanism that very cleverly lightweights mhsa through operations such as sparse matrices, lightweight convolution, and so on, without reducing accuracy. Please add the cleverness of biformer and reduce the description of mhsa, because mhsa has become common knowledge in the field of attention mechanisms.

Response:

Thank you for your suggestion. We have added relevant descriptions in the fourth paragraph of section 2.3:

“a two-layer routing attention mechanism is utilized to filter out most of the irrelevant K-V pairs in the coarse-grained region, followed by applying token-to-token attention to focus on a small portion of the relevant tokens, which provides good performance and computational efficiency since it does not distract the attention of irrelevant tokens.”

4. Please note the specification of equation 16, which uses "F1" instead of "F". In addition, there are formatting errors in the two lines of text below equation 16, such as font and spacing. Please correct them carefully.

Response:

We have replaced F1 with F and modified the format of these lines.

5. In Section 4.4, "The results for training accuracy, recall, and F-value are shown in Table 1." does not match the performance metrics in Table 1, so if English is not the first language, please check after using the translator. The article is written with a terrible attitude. Moreover, why "training accuracy"? Why not use test set results?

Response:

We apologize for making such a low-level error and sincerely appreciate your comments and questions.

(1) We have changed F-value to F-measure and double-checked the whole text to correct word-correlation errors again.

(2) Why "training accuracy"? Why not use test set results?

In order to ensure the rigor of the scientific paper and the scientific validity of the experimental results, we follow your suggestion. Replace the Table1 experimental data with the test set and add relevant descriptions in section 3.4: ”The test results of Precision, recall, and F- Measure are shown in Table 1.”

6. Why are "Params" in table 2 and table 4 not using the same unit?

Response:

We have changed “model size(M)” to “Params(M)” in Table 2.

7. There may be problems with the two sets of experiments in Table 3. How are "vit+inference block" and "swin-t+inference block" completed? The "inference block" is a variant of mhsa. vit and swin-t are also variants of mhsa. Therefore, how the experiments are completed? Please give a reasonable explanation.

Response:

(1) The root of the problem is that you feel reusing MHSA modules will not complete the experiment. We believe that as long as upstream (2D image features) and downstream (1D sequence features) tasks processed with MHSA are different, the stacking is not considered invalid. The representative model that validates our theory is NRTR (A No-Recurrence Sequence-to-Sequence Model For Scene Text Recognition, [https://doi.org/10.48550/ arXiv.1806.00926](https://doi.org/10.48550/%20arXiv.1806.00926)） publishing in CVPR in 2019. The NRTR encoder uses stacked self-attention to extract image features and the decoder applies stacked self-attention to recognize text based on the encoder output, relying only on the self-attention mechanism, which can be trained with higher parallelism, and achieves the best performance.

(2) Back to the experiment in this paper, we use VIT/Swin to extract visual features, then use the inference block to focus on characters that will result in sequential features, and finally do localization through the loss function CTC. The application of the task and feature dimensions are different, so the experiment can be completed.

8. Why is the cpu used in Table 4? what does the gpu do in the experimental environment? Please give a reasonable explanation.

Response:

(1) Why is the cpu used in Table 4?

CPU is only used in Tables IV and V for inferencing. Comparing on lightweight models, CPU better shows the speed difference, considering that cpu is used more widely in future scenario applications.Our LSTR is a lightweight model targeting devices with CPU only.

(2) what does the gpu do in the experimental environment?

GPU is used in Tables I, II, III, and V to train the model.

9. Why are the performance metrics described differently in each table? This is very unfriendly to reviewers and readers.

Response:

Thank you very much for raising this fatal issue for us. There are some serious mistakes sloppy formulations in the writing process. We have now improved Table 2,4,5 by standardizing the model parameter count size to params (M) and the inference speed to FPS or speed (ms). The evaluation criteria of detection and recognition models are often different, FPS for detection and ms for recognition.

10. The performance metrics of the comparative models in Table 5 are too few, so please add the results.

Response:

Thanks to your suggestion, we have added a comparison of “Params(M)” in Table V.

11. It's a well-known fact that the fastest way to get academic resources is not to work hard, but to just loot them. CV field ≈ ctrl c + ctrl v field.

Response:

We apologize and feel great guilt for making some low-level mistakes, such as inconsistent experimental standards and inaccurate translations, which may have brought you an unpleasant experience in reviewing the manuscript. However, from determining the research topic, manually collecting datasets and labeling them, to completing each part of the experimental code and parameter deployment have gathered a lot of our efforts. For example, fc_decay=0.004, is the optimal result after more than 1 month of experimentation.

Finally, we thank you for your kind reminder, and we will ensure that the experimental data are rigorous and scientific.

12. The problem of seal information localization and recognition belongs to the problem of small target recognition, but the research on such aspect in these paper are clearly insufficient, for example, the achievement in these two literature have not even been mentioned: DOI:10.1093/jcde/qwac071,DOI:10.1007/s10462-023-10438-y,and so on.

Response:

We are sorry that we have previously focused only on the field of text recognition and detection. After carefully reading these two articles about small target recognition we feel that they are very applicable to the scenario of this paper and have added citations and relevant descriptions in the fourth paragraph of the introduction：“In addition, by combining cross-stage feature fusion and attention mechanisms[10,11], designing lightweight CNN can also improve accuracy for small target detection and recognition“

13. Therefore, please draw your own diagram of the general architecture of the model, not the DBNet prototype, so that the subsequent image structure is represented in the general architecture diagram.

Response:

Although displaying an image of the original seal may result in copyright infringement,we have taken your advice and proceeded to draw our own DB-ECA model as shown in Figure 2, and added the relevant notes.

Fig 2. DB-ECA model diagram, where “pred” consists of a 3×3 convolutional operator and two de-convolutional operators with stride 2. The “1/2”, “1/4”, ... and “1/32” indicate the scale ratio compared to the input image.

**To reviewer #2**

1. The author proposes his own method for the detection and recognition of Chinese seal text, which has practical significance. The overall article is innovative and has sufficient workload.

Response:

Thank you very much for recognizing our research!

2. The word detection section can be compared with other algorithms, including classic text detection algorithms based on regression methods. Conduct ablation experiments on the improved part and add more comparative images of experimental results.

Response:

(1) At your suggestion, we added a comparison of DB-ECA with other segmentation- or regression-based (East, TextBoxes++) models and added relevant descriptions in the Revised Manuscript in second paragraph of section 3.5.

Figure 8 Detection results of different models. The number in the center of the bubble indicates Precision, and the area of the bubble is proportional to F-Measure.

(relevant descriptions)“In Figure 8, we can see that TextBoxes++[30] requires a large number of datasets for model pre-training due to the complex network structure, and the low-level feature expression is weak, CTPN[31] joins the LSTM in the training phase which easily leads to gradient explosion, and it cannot process multi-directional text, so they are not suitable for small-scale target detection such as the seal in this paper, and their precision are respectively lower than those of DB-ECA by 6.71% and 7.63%.Although DRRG can realize the detection of arbitrary shaped text, the detection results are overly dependent on the individual word detection frames suggested by the text component.PRPN[32] proposes a two-dimensional asymptotic kernel to satisfy the requirements of the curved text detection task, but there are difficulties in dealing with text embeddings like seals and other rare training data, and thus the Precision is lower than that of DB-ECA by 4.96%, respectively, 3.48%. The experimental results demonstrate that DB-ECA achieves the highest Precision and F-Measure while maintaining a high FPS”.

(2) In fact, we have demonstrated the effectiveness of adding ECA and improving ResNet by ablation experiments in Table 1, in order to verify its authenticity. We have added more comparative pictures of the experimental results, such as the heat map.

（a）

（b）

Heat map comparison results（a）is the heat map presented by the original DBNet module after the cascade of features，(b) is the heat map presented after adding ECA and improving ResNet

3. It should be explained whether seal images with completely opposite text directions can be detected and recognized.

Response:

(1) Our model can recognize and detect stamps with opposite orientations. Our training dataset has stamps in opposite directions, as showing below

(2) Due to the inclusion of random rotation, our model is able to detect and recognize the opposite direction of the stamp text, but the extracted text is read from left to right by default. It may bring reading obstacles, so we provide additional clarification in section3.1：“Adding analog simulation effects such as rotation and transparency in the production of electronic seals to enhance the training effect and generalization ability of the model for seals with different text orientations.”

4. The data volume is not large enough, and more seal images can be collected or more dataset images can be obtained through transfer learning and other methods.

Response:

(1) We have trained on the recognition model using transfer learning and illustrate it in the third paragraph of section3.4: “Since recognizing characters requires a large number of real datasets more, we pre-train LSTR on the public dataset CTW (Chinese Text in the wild) for 100 epochs by transfer learning, and then train it on the homemade dataset.”

(2) Electronic seals(c)(d) are far from real seals(a)(b), and making more electronic seals can even have a negative effect on the model. Real seals are a representation of the rights of the relevant authorities and are very difficult to collect. Therefore, we consider transfer learning to solve the problem of lack of datasets.

.

（a） （b）	（c）	（d）

The topic of this manuscript is to improve the accuracy of Chinese seal recognition. In my opinion, there are two innovative points in this paper. Firstly, a model DB-ECA was constructed to improve the differentiable binarization detection algorithm (DBnet). Secondly, a model named LSTR (Lightweight Seal Text Recognition) was propose for text recognition. In my opinion, the method used in this paper is innovative, but the application area is not very attractive. Therefore, I suggest that the manuscript can be published after major revision. Before acceptance for publication, the paper needs following improvement:

**To reviewer #3**

1. Line 10-12. The specific significance of this study should be supplemented in the beginning of the abstract. Which fields or works will this research be most helpful for? What is the important significance of extracting text information from Chinese seals?

Response:

Relevant notes have been added in the beginning of the abstract: ‘Recognition of the key text of the Chinese seal can speed up the approval of documents, and improve the office efficiency of enterprises or government administrative departments.’

2. Line 18-23. If this journal does not limit the word count of abstract, I suggest supplementing the results and conclusions of this study in the abstract.

Response:

Relevant notes have been added in abstract:” Experiments on the homemade dataset in this paper, DB-ECA compared with the other five commonly used detection models, the precision, recall,F-measure are the best effect of 90.29,85.17,87.65, respectively. LSTR compared with the other five kinds of recognition models in the last three years, to achieve the highest effect of accuracy 91.29%, and has the advantages of a small number of parameters and fast inference. The experimental results fully prove the innovation and effectiveness of our model.”

3. Line 25-29. I suggest that the author add the content of the significance and importance of this study at the beginning of the introduction

Response:

Relevant notes have been added at the beginning of the introduction: ”Seal is used to print on the document to indicate identification or signed stationery, because of its production is simple and distinctive signs, widely used in government, enterprises and other organizations issued documents. Although the production methods and styles are not uniform in different countries, most of them have legal effect and occupy an important position in the administrative office.”

4. Line 73. Can this section “2.Related Work” be merged with the introduction? Most studies would analyze the methods and results of previous research in the introduction. If it can be merged into Introduction, I suggest rewrite the content of this section. The introduction should be reduced to 5-6 paragraphs with a summary at the end of each paragraph.

Response:

We have followed your suggestion to merge the related work into the introduction, introduce and compare the methodology of this paper with the related work, delete the original line139-143,reduce the repetition of the methodology. You can see the changes in the revised introduction in blue.

5. Line 146-158. A title should be supplemented before the text of this paragraph？

Response:

Replenished in section 2.1 : “2.1 Overall structure”

6. Line 158-160. Is Figure 1 original by the author? Can the first input small picture be replaced with the pictures related to this study? I suggest that the title of this figure be written in more detail. It is necessary to add descriptions of important parts and some small figures.

Response:

(1) Sorry Figure 1 is not the author's original, it is the original DBnet[15] picture.

(2) Due to possible infringement of the original seal image.We have plotted Figure 1: DB-ECA, ourselves figure and replaced the input, binarized heat map, and output images with our dataset as follows：

Fig 1.DB-ECA overall structure,where “pred” consists of a 3×3 convolutional operator and two de-convolutional operators with stride 2. The “1/2”, “1/4”, ... and “1/32” indicate the scale ratio compared to the input image.

7. I strongly suggest that the author can supplement a diagram as Figure 1 to introduce the overall process of this study.

Response:

We have followed your suggestion to add the general structure diagram and related description in section2.1:

“Our proposed method as a whole can be divided into two parts, (1) using segmentation-based suitable for curved text of the differentiable binarization algorithm to construct the detection model DB-ECA, and improve it, which can reduce the workload of preprocessing and streamline the model structure (2) Propose a lightweight seal recognition model LSTR, which can achieve high recognition accuracy for fuzzy, deformed and other irregular Chinese seals without collecting a large number of real seal datasets by using CNN's inductive bias and self-attention. The overall structure of this paper is shown in Fig. 1.”

Fig 1.General structure of the paper

8. I suggest merging Figures 2 and 3 into one figure and displaying them separately with panel (A) and (B). In addition, the styles and colors of the two small figures should be consistent.

Response:

Fig 2: (A)BottleNeck structure diagram(B)ECA structure diagram

9. Are the three text recognition results in Figure 7 are screenshots of a programming tool? I don't like the style of this figure. Readers outside China cannot understand its meaning. I suggest that the Figure 7 should be redraw. The small pictures of the 3 seals and their text recognition results should be divided into 3 different groups and panels. The figure caption should describe the three panels in details.

Response:

In order to emphasize the authenticity of the experimental data, our previous recognition effect in Fig 7 is a screenshot of the output of the prediction results in the programming tool. According to the requirements of the journal, the display of the original image of the seal may cause infringement, so we apologize for the deletion of Figure 7.

10. Line 145-301. I suggest reducing at least 30 lines text of “Text detection and recognition model”. I think the description in this section is not concise, and some redundant content should be reduced.

Response:

At your suggestion, we have reduced unnecessary descriptions by deleting the line 139-143 in the original text and removing first paragraph of section 2.2 from the original text.

However, according to the suggestions of two other reviewers, the content has been supplemented. Therefore, "Text detection and recognition model" is still about 150 lines.

11. The paper is generally understandable. It is suggested the author check carefully the English writing and use standard terminologies in the neural network areas.

Response:

Thanks to your suggestion, we double-checked the full text and replaced some of the expressions with neural network fields. For example, we changed “mode size (M)” to “Params (M)” in Table II, “migration learning” to “transfer learning” in third paragraph of section3.4,and ”microscopic binarization” to “differentiable binarization” in “main contributions”.
